# Antibiotic Resistance in Aquaculture: Challenges, Trends Analysis, and Alternative Approaches

**DOI:** 10.3390/antibiotics14060598

**Published:** 2025-06-11

**Authors:** Elshafia Ali Hamid Mohammed, Béla Kovács, Ronald Kuunya, Eltayeb Omaima Awad Mustafa, Azza Siddig Hussien Abbo, Károly Pál

**Affiliations:** 1 Department of Animal Husbandry, Institute of Animal Science, Biotechnology and Nature Conservation, Faculty of Agricultural and Food Sciences and Environmental Management, University of Debrecen, 4032 Debrecen, Hungary; 2 Doctoral School of Animal Science, University of Debrecen, 4032 Debrecen, Hungary; 3 Agricultural Research Corporation, Integrated Pest Management Research Center, Wad Madani 21111, Sudan; 4 Institute of Food Sciences, Faculty of Agricultural and Food Sciences and Environmental Management, University of Debrecen, 4032 Debrecen, Hungary; kovacsb@agr.unideb.hu (B.K.); pal.karoly@agr.unideb.hu (K.P.); 5 Institute of Land Use, Engineering and Precision Farming Technology, Faculty of Agricultural and Food Sciences and Environmental Management, University of Debrecen, 4032 Debrecen, Hungary; kuu79ron@mailbox.unideb.hu; 6 Department of Public Health and Epidemiology, Faculty of Medicine, University of Debrecen, 4028 Debrecen, Hungary; omaima.awad@med.unideb.hu; 7 Department of Crop Protection, Faculty of Agriculture, University of Khartoum, Khartoum North 11115, Sudan; azza.abbo@gmail.com; 8 Ministry of Municipality, Doha 00000, Qatar

**Keywords:** aquaculture, antibiotic resistance, antibiotic resistance genes (ARGs), bibliometric analysis, research trends, R software

## Abstract

Antibiotic resistance in aquaculture has emerged as a global crisis, representing a serious threat to the health of aquatic animals, environment, and human. The extensive use of antibiotics in aquaculture has led to rapid development of resistant bacterial strains, resulting in environmental contamination and the dissemination of resistant genes. Understanding of the research trends, key contributors, and thematic evolution of this field is essential for guiding future studies and policy interventions. The study aimed to conduct a bibliometric analysis of research on antibiotic resistance development in aquaculture, identifying key areas of research, leading contributors, emerging challenges, and alternative solutions. Data were extracted from the Web of Science (WoS) database covering the period from 2000 to 2025. A systematic search strategy was employed, utilizing terms including “antibiotic resistance” AND “bacteria,” AND “aquaculture”. Relevant publications were extracted from the WoS using these keywords. R-tool was then used to analyze the obtained metadata including keywords, citation patterns, and co-authored country. The analysis revealed a remarkable increase in publications over the past 25 years, with key contributions from China, India, and the USA. The most significant articles focused on the presence of multidrug resistant bacteria in the aquatic environments and, antibiotic-resistant genes, and horizontal gene transfer. Probiotics are the alternative solution to overcome the antibiotic resistance and enhance aquaculture sustainability. Future research should focus on the interdisciplinary collaboration, novel antimicrobial alternatives, and global monitoring approaches.

## 1. Introduction

Aquaculture is among the most rapidly growing animal industries, contributing approximately 94 million tons of aquatic animals and providing 15% of animal protein [1]. It plays a considerable role in the global food supply [2,3]. The global aquaculture industry has shown a consistent annual growth rate of 5.8% since 2001, reflecting the increased animal protein demand in rapidly growing economies. Aquaculture sectors have been largely driven by Asia, which accounts for around 90% of global aquaculture production, with China contributing more than 61% of the total in 2016 [4]. However, the presence of pathogens such as *Aeromonas hydrophila*, *A. salmonicida*, *Vibrio harveyi*, *V. anguillarum*, *Pseudomonas fluorescens*, *Flavobacterium psychrophilum*, *Yersinia ruckeri*, and *Citrobacter freundii* has been observed and may adversely affect the production of fish and other aquatic animals [5].

Antibiotics are extensively utilized in aquaculture to manage bacterial pathogens and improve fish performance [6,7,8]. Low levels of antibiotics in feed have been used in many countries to promote growth performance. Several studies have indicated that the administration of antibiotics such as oxytetracycline, chloramphenicol, and florfenicol might have a growth-promoting effect on cultured tilapia (*Oreochromis niloticus*) [9,10]. However, the continuing use of aquaculture antibiotics has enhanced the natural selective pressures on microbial communities, leading to the emergence of antibiotic-resistant strains that are capable of widespread dissemination and causing severe infections [11]. These resistant bacteria can negatively impact aquaculture production, fish consumers, and the aquatic environments [12,13,14]. Consumption of farmed fish contaminated with antibiotic-resistant bacteria can result in public health consequences [15]. Because of their unique chemical structure, antibiotics are difficult to break down or degrade in the natural environment and can easily circulate in the food chain and accumulate in the human body [16]. The rapidly increasing number of antibiotic resistance genes (ARGs) found in the aquaculture environments is one of the major public health problems associated with the use of antibiotics [17,18,19]. In 2019, it was estimated that serious infections caused by antibiotic-resistant bacteria directly caused more than 1.27 million deaths worldwide and contribute to an additional 4.95 million deaths [20]. Antibiotic-resistant bacteria can transfer resistance to antibiotics and reduce the effectiveness of antibiotics in the aquatic systems in which they proliferate, and the consumption of farmed fish contaminated with antibiotic-resistant bacteria can pose a public health concern [21]. Additionally, ARGs can rapidly spread to other bacterial species by horizontal gene transfer [22,23].

Bibliometric methods are considered to be a powerful tool to evaluate and assess qualitative data and quantitative information about research activities [24]. Bibliometric analysis involves searching databases and using statistical methods and mapping techniques to perform quantitative and qualitative analyses of specific bibliometric parameters [25]. By extracting data from paper titles, affiliations, abstracts, keywords, collaborative networks, and trend characteristics, it is possible to construct and identify quantitative trends [26,27]. Recently, researchers have effectively analyzed research hotspots and trends in/their respective fields using bibliometric tools and information, such as authorship, institution, country, co-citation analysis, and co-authorship analysis [28,29,30,31,32,33]. For this reason, we chose this technique for our study to analyze future research directions related to the antibiotic-resistant bacteria in aquaculture.

Several studies have been published previously on different aspects of antibiotic resistance (ABR), such as ABR in natural waters [34], soil [35], animal intestines [36], fish farms and ornamental fishes [37,38], and the global shrimp industry [39]. However, systematic reviews focusing on the impact of antibiotic resistance in aquatic environments are still few. Consequently, the current study aimed to provide a comprehensive bibliometric analysis of trends in the consequences of aquaculture antibiotics. Specifically, we aimed to (1) identify the structure, volume of the global scientific literature, and highlight research gaps in the field of AMR in global aquaculture, (2) focus on the destructive potential of antimicrobial resistance in aquaculture, and (3) discuss the future strategic directions and sustainable solutions to antibiotics.

## 2. Materials and Methods

### 2.1. Research Questions

(a) What are the main areas of research, volume of research, geographical distribution of the literature, co-authored countries, trends, the significant papers, and leading journals on the studies that focused on antibiotic-resistant bacteria in aquaculture (ABA)? This investigation was based on a selection of keywords, including “antibiotic resistance” AND “bacteria” AND “aquaculture”.

(b) What are the specific consequences of antibiotic-resistant bacteria on fish health and aquatic ecosystems?

(c) What are the alternative strategies that can be employed to overcome or at least reduce the occurrence of antibiotic resistance in aquatic environments (with a focus on probiotic bacteria)?

### 2.2. Framework and Searching Strategy

Bibliometric-based studies are typically performed by examining global literature from the Web of Science (WoS) database. The WoS has been selected because it is considered the world’s most prominent database for the evaluation and analysis of scientific research, encompassing the most significant and impactful research outcomes on a global scale [28,40]. The search was conducted on 5 April 2025, using a combination of keywords including antibiotic resistance, bacteria and aquaculture, with a time frame extending from 2000 to 2025. All documents that were originally written in English were included. Moreover, all open access published research articles, conference papers, and book chapters were considered, while other types of literature were excluded. The search generated a large number of documents. To achieve the first objective (Objective a), a search was performed on the WoS database based on titles, affiliation, abstracts and keywords. Obtained metadata, which comprised 1869 documents that contained all keywords, were then considered for bibliometric analysis and reviewing.

### 2.3. Bibliometric Analysis

The bibliometric information, including authors, document titles, types of documents, countries of affiliation, publication dates, author keywords, and publishing entities, was exported as *BibTeX* format from the WoS database. We employed R-Studio v. 2024.04.1+748 (PBC, Boston, MA, USA) in connection with the bibliometric R-package to perform in-depth analyses, including citation analysis, co-occurrence keyword assessments, coupling mapping, factorial analysis, network algorithm, and multiple correspondence analysis. The flexibility and statistical capabilities of R software are essential to effectively visualize the large amount of complex data [41]. Briefly, the obtained metadata (bib data) that were exported from WoS were converted into *BibTeX* format, and then the bib data were uploaded to the R-bibliometric package for comprehensive bibliometric analysis [41]. This analysis yielded multiple knowledge maps that illustrate the nature of the emerging research in the field of antibiotic-resistant bacteria in aquaculture. Then, the obtained results and summary of the statistics were exported to Microsoft^®^ Excel 2016 v. 16 (Microsoft Corporation, Redmond, WA, USA) for further processing and tabulation. The results were then visualized as clusters to identify the key areas of possible research gaps and limitations in knowledge about the geographical regions (countries) in which the studies were carried out.

### 2.4. Screening and Extraction

**Figure 1:**
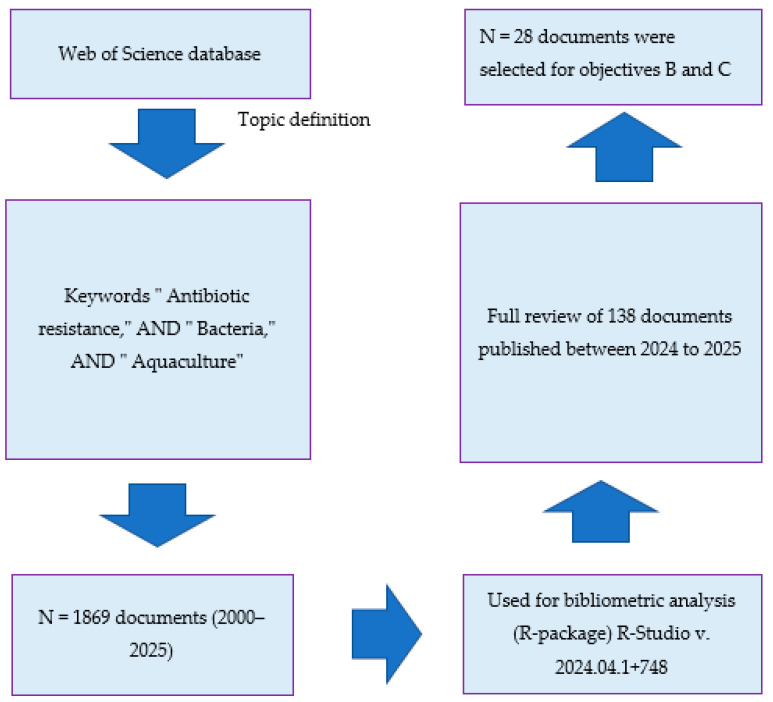
Workflow of ABA documents’ screening and processing on the obtained metadata from web of science (WoS) database (2000–2025). ABA: “antibiotic resistance”, “bacteria”, and “aquaculture”.

Regarding Objective “b”, the research articles published between 2024 and 2025 were reviewed, and the information that outlined the destructive potential of antibiotic-resistant bacteria was selected, summarized and discussed (Figure 1). Briefly, data including country, antibiotic-resistant bacteria, aquatic animal host, antibiotic group, and resistant gene/s were extracted manually or by using Web Plot Digitizer v. 5.0 (Ankit Rohatgi, Austin, TX, USA), where data were presented as figures. The same screening method was used for Objective “c”. However, data including country, probiotic species, aquatic animal, and main outcomes were considered.

## 3. Results and Discussion

### 3.1. The Situation of the Scientific Publications Based on WoS Search on Antibiotic Resistance, Bacteria, and Aquaculture (ABA)

**Figure 2:**
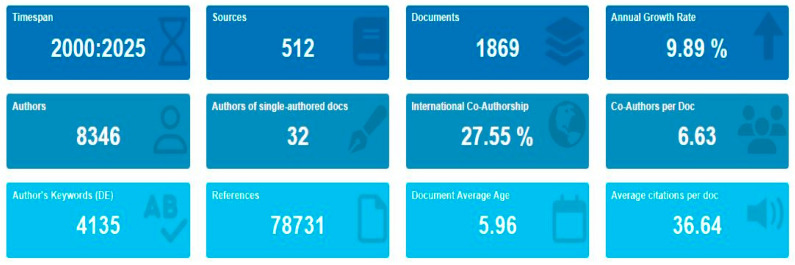
Overview of bibliometric indicators for published ABA documents from 2000 to 2025. Metrics include the number of sources, papers, authorship patterns, citations, international collaboration, and keyword diversity. ABA: “antibiotic resistance”, “bacteria”, and “aquaculture”.

A general overview of the bibliometric dataset is presented in Figure 2. In total, 1869 documents for the period between 2000 and 2025 were found in 512 sources (e.g., journals, conferences, proceedings, and books). The annual scientific production exhibits a notable annual growth rate of 9.89%, indicating increasing research interest in the field over the past two decades. The publications were authored by 8346 authors, with a relatively low number of single-author publications (32 authors), suggesting a strong culture of collaboration. The average document had 6.63 co-authors, and international co-authorship was involved in 27.55% of the analyzed documents, highlighting the global nature of research in the area of ABA.

#### 3.1.1. Growth of Scientific Publications of ABA Documents

**Figure 3:**
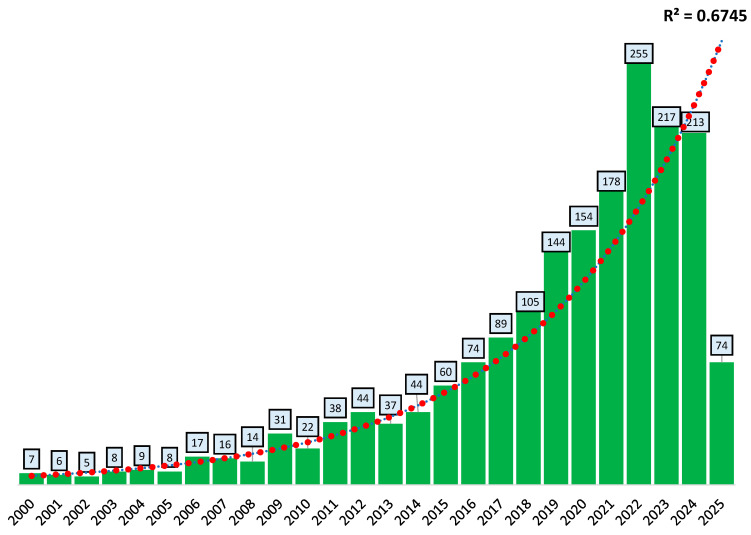
Annual growth of scientific publications of ABA documents based on the WoS database. The correlation coefficient (R^2^) of the exponential curve between 2000 and 2025 was 0.925. ABA: “antibiotic resistance”, “bacteria”, and “aquaculture”.

According to the WoS, the ABA documents between 2000 and 2025 have yielded considerable attention. These documents, with the number of publications (n) and percentage (%) included, are as follows: research articles (n = 1568, 83.9%), review papers (n = 282, 15.1%), conference proceedings (n = 33, 1.8%), and book chapters (n = 22, 1.2%). Figure 3 illustrates the annual growth of scientific publications of ABA documents from 2000 to 2025. The number of publications remained relatively low and stable between 2000 and 2007, with fewer than 20 documents per year. However, since 2008, a remarkable upward trend started, with a sharp increase in publication outputs after 2015. The highest output was recorded in 2022 with 255 publications, followed by 217 in 2023 and 213 in 2024. The noted drop in 2025 (n = 74 publications) is likely due to the partial nature of the year at the time of data collection (April 2025).

A trend line was calculated with an R^2^ value of 0.9251, indicating that the progress curve of the ABA documents followed an exponential trend. This strongly suggests a consistent and exponential increase in ABA research interest and activity. The data reflect a maturing and expanding body of literature, potentially driven by growing academic, technological, or societal interest in this research topic. This could indicate a significant increase in ABA research over the past two decades, demonstrating the global concern about antibiotic resistance in aquaculture. According to the projections, the world’s population will exceed 9 billion by the year 2050, and food production is expected to increase by more than 85% [42]. To address this issue, aquaculture antibiotics are extensively used to maximize the productivity of aquatic species. For example, according to the findings reported by Lulijwa et al. [43]. Approximately 73% of the major aquaculture-producing countries have been documented to utilize antibiotics such as oxytetracycline, florfenicol, and sulfamethoxazole. Additionally, 55% of these nations have been observed to employ erythromycin, amoxicillin, sulfamethoxazole, and enrofloxacin in their aquaculture practices.

#### 3.1.2. Country-Level Scientific Collaboration for ABA Research

The top 20 most productive countries in terms of ABA scientific publications are presented in Table 1. China is in the lead with 520 publications, accounting for 27.82% of the total output (1869 documents). India ranked second place with 203 publications, accounting for 10.86% of the total research volume of ABA, and Malaysia is in third place with 77 publications, accounting for 4.12% of the total ABA documents. The analysis reveals that these three countries collectively account for over 42% of the total scholarly output, indicating the dominance of Asian countries in this area of research.

In addition, single-country publications (SCP) and multiple-country publications (MCP) are considered key indicators of international collaboration (Table 1). It is noteworthy that while China exhibits the highest overall productivity, its international collaboration rate (MCP ratio = 15%) was relatively low. In contrast, countries such as Thailand (62.16%), the United Kingdom (47.83%), Japan (48.15%), and Belgium (51.72%) exhibit a high degree of international collaboration, with more than 45% of their publications co-authored with researchers from other countries. This result suggests that while certain countries generate a substantial volume of research independently, others may prioritize global partnerships to amplify research impact and facilitate knowledge exchange. Malaysia, Spain, and Egypt reflect a significant international collaboration trend, with MCP ratios above 38%.

**Figure 4:**
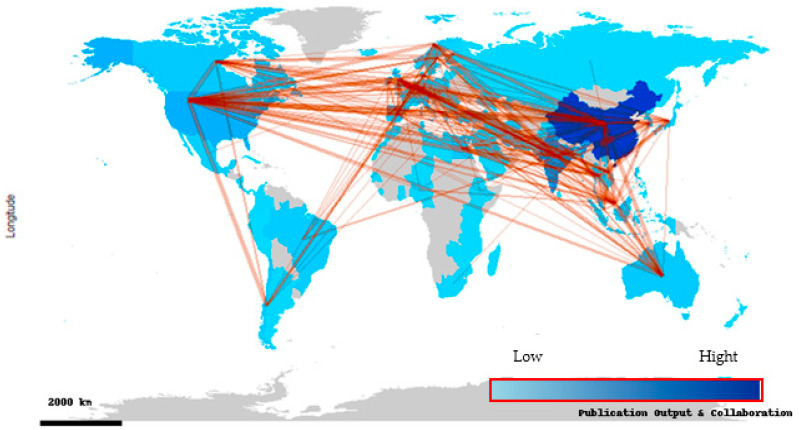
The map illustrates international collaboration patterns based on co-authorship in publications related to antibiotic resistance in aquaculture (2000–2025). Countries are color-coded by publication output, with darker shades indicative of higher productivity. The connecting lines represent collaborative links between countries, and their thickness corresponds to the intensity of co-authorship.

The international collaboration network among countries contributing to the antibiotic-resistant bacteria in aquaculture (ABA) is presented in Figure 4. The global map visually represents the intensity of collaborations and geographic spread of ABA documents, with nodes representing countries and connecting lines (edges) indicating co-authored countries across borders. The most prominent country in this network was China, highlighted in dark blue, reflecting both its high publication output and extensive international collaboration (Figure 4). China is the largest producer and exporter of aquatic products in the world, and is also a major user of antibiotics in aquaculture [44]. In 2017, China consumed 57.9% of the world’s antibiotics and produced 51.2% of the world’s aquaculture production [45].

In addition, China appeared to collaborate frequently with different countries across North America, Europe, South America, Southeast Asia, and Africa, suggesting a central role in global scientific cooperation within this research area. Other prominent collaborating countries are the USA, the United Kingdom, India, Malaysia, and Spain. All of which show multiple connecting lines to diverse regions. Africa and parts of South America also participate in the global research network for ABA documents, albeit with fewer links, indicating growing but still limited international engagement. The density and direction of the collaboration lines illustrate a well-connected global research community. Strong ties between Asian and European countries, as well as increasing links between Asia and the Americas, emphasize the international and interdisciplinary nature of the ABA research field. This map confirms the findings from the country productivity table, where countries with high MCP ratios (e.g., Thailand, the United Kingdom, and Belgium) also appear to be well integrated into the global network. Such collaboration is crucial for advancing the complex and globally relevant research topics.

**Figure 5:**
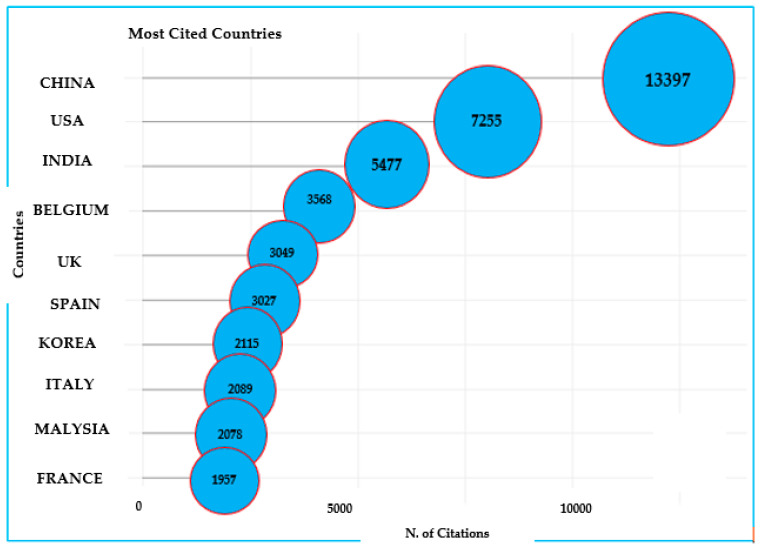
The top 10 most cited countries on ABA documents (2000–2025) based on WoS. ABA: “antibiotic resistance”, “bacteria”, and “aquaculture”.

Furthermore, the top three most cited countries in the ABA research field were China, USA, and India (Figure 5). China leads by a significant margin with 13,387 citations, indicating its substantial role and extensive contributions to this field. The United States follows with 7226 citations, reflecting its continued influence in global research. India, Belgium, and the United Kingdom round out the top five, each contributing between 4000 and 6000 citations. Notably, European countries such as Belgium, the United Kingdom, Spain, and Italy also appear prominently, suggesting strong academic engagement across the region. The presence of Korea, Malaysia, and France further emphasizes the global interest and collaboration in this area of research. Overall, the results highlighted the international nature of scientific contributions and the leadership of China and the United States of America in the citation record.

The substantial increase in China’s research output has led to its emergence as a prominent player in the global academic publishing landscape, including the publication of studies related to antibiotic resistance in aquaculture. All of this together indicates that China now exceeds the United States and any other country in terms of the number of researchers and scientific outputs, and the quality of these outputs has improved significantly, as evidenced by the rising number of citations in top-ranked journals [46].

#### 3.1.3. The Journals, Publishers, and Most Cited Publications on ABA Documents

Table 2 presents the most cited papers and the top 20 most prominent journals in the field of ABA based on the WoS database. The citation analysis of the most cited publications reveals significant trends in research impact and popularization in environmental microbiology and related disciplines. The paper by Cabello F.C. (2006) ranked first by 1607 citations and 80.35 citations per year, highlighting its foundational impact in the field of antibiotic resistance in aquaculture. Remarkably, despite being one of the most recent publications, Zainab S.M. (2020) has the highest citation rate per year (104), highlighting the growing urgency and relevance of current issues such as emerging contaminants. Environmental Pollution and Water Research, both published by Elsevier, are prominent and appear multiple times, indicating that these journals are important dissemination platforms for high-impact work. In addition, emerging publishers such as MDPI and Frontiers Media have become increasingly important following the recent papers, namely Watts JEM (2017) (438 citations) and Vieco-Saiz N (2019) (366 citations), suggesting the rise in open access publishing platforms in increasing visibility and citation potential.

### 3.2. The Destructive Potential of Antibiotic-Resistant Bacteria and ARGs in Aquaculture Based on Keyword Analysis

#### 3.2.1. Co-Occurrence Network of Keywords

**Figure 6:**
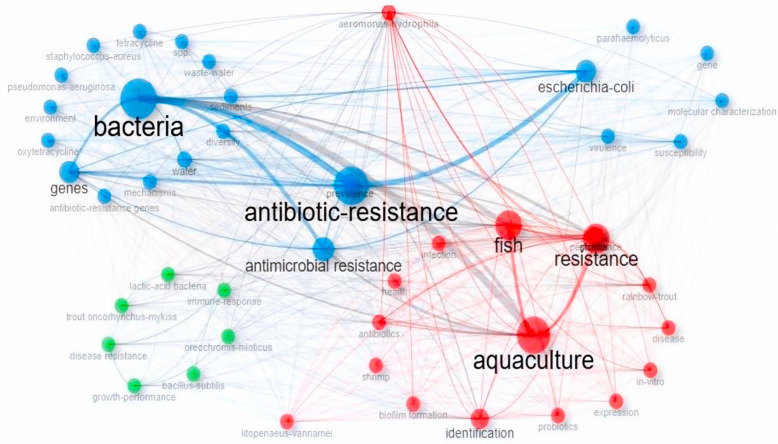
Keyword Co-occurrence Network Based on Author Keywords: The size of each node represents the frequency keyword occurrence. Different colors indicate clusters of closely related keywords for ABA documents based on WoS database (2000–2025). ABA: “antibiotic resistance”, “bacteria”, and “aquaculture”.

The result of the keyword co-occurrence network is shown in Figure 6. The most frequently used terms (larger nods) and their interrelationships within the literature were antibiotic resistance, bacteria, aquaculture, fish, and *Escherichia coli* (*E. coli*), respectively. The network is organized into distinct clusters, each representing a thematic research area. The red cluster, with keywords such as aquaculture, fish, and resistance, reflects research focused on aquaculture practices and resistance issues in aquatic organisms. The blue cluster revolves around bacteria, antibiotic resistance, and *Escherichia coli*, indicating a strong focus on bacterial pathogens and antimicrobial resistance mechanisms.

Notably, *E. coli* emerges as a central term within the blue cluster, emphasizing its critical role both as a model organism and a pathogenic indicator in aquaculture-related microbiological studies. Its frequent co-occurrence with terms like antibiotic resistance, virulence, and molecular characterization suggests that *E. coli* is often studied in the context of resistance gene transfer, environmental contamination (e.g., from wastewater), and health risk assessments in aquatic ecosystems. The presence of *E. coli* in aquaculture environments raises concerns about fish and human health and highlights broader implications for environmental antibiotic resistance proliferation.

#### 3.2.2. The Most Frequent Keywords over Time

**Figure 7:**
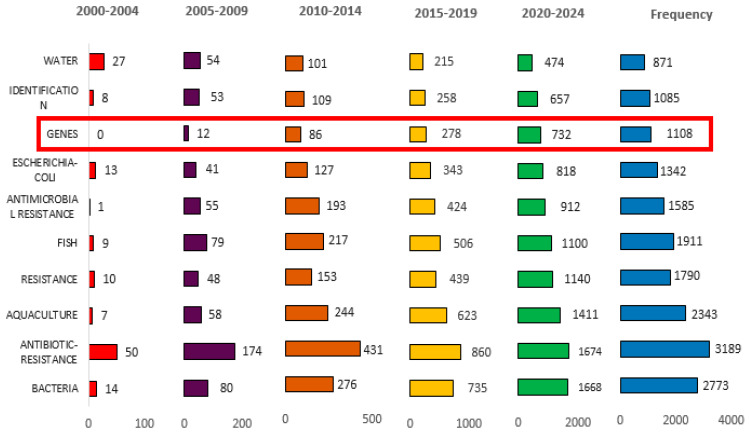
The most frequently used in keywords on ABA documents over five-consecutive years, (2000–2025). ABA: “antibiotic resistance”, “bacteria”, and “aquaculture”. The red box shows the keyword “genes”. It illustrates the substantial increase in usage over the years.

The results of the search for the most frequent keywords over time across five-year intervals from 2000 to 2024 are displayed in Figure 7. In the early 2000s (2000–2004), the most repetitive terms included “antibiotic resistance” (50 mentions), “water” (27 mentions), and “bacteria” (14 mentions), reflecting a core of interest in identifying microbial threats and characterizing resistance in basic terms. However, by the next interval (2005–2009), research had begun to intensify, with notable increases in terms like “antibiotic resistance” (174 mentions), “fish” (79 mentions), and “aquaculture” (58 mentions). This could reflect a growing awareness of aquatic environments as a critical source for antimicrobial resistance development and transmission, particularly in the context of food production systems. In 2006, Akinbowale et al., [63] reported that increased incidence of serious infections and treatment failures have been reported in humans as a result of the transfer of antibiotic resistance from aquaculture to humans through the diet associated with aquaculture products.

From 2010 onwards, the rate of increase across all major keywords becomes more prominent. For instance, “antibiotic resistance” recorded 431 mentions (2010–2014), then 860 (2015–2019), and 1674 (2020–2024). Similarly, “bacteria” rises to 276, 735, and 1668 mentions, respectively, over the same periods. These high increases align with intensified global concern regarding antimicrobial resistance as a public health disaster, highlighted by initiatives such as the WHO’s global action plan on antimicrobial resistance [64].

A notable pattern is the rise in “genes” as a research keyword, which had no mentions in 2000–2004 (Figure 7). However, rapidly increased to 278 (2015–2019) and 732 (2020–2024). This indicates a major technological shift toward genomic and metagenomic technologies, enabling researchers to identify resistance factors, mobile genetic elements, and bacterial community structures with extensive information. Metagenomic technologies have emerged as crucial tools for investigating antibiotic resistance in aquatic environments, addressing the diversity of microbial communities and the dissemination of resistance genes and toxic proteins [65,66,67]. The ability of metagenomics to overcome the limitations of culture--dependent methods facilitates the detection of a vast range of ARGs, including those harbored by non-cultivable organisms, thereby offering nuanced insights into the structure and function of microbial communities in aquatic environments [67]. In this regard, metagenomic-based methods enabling not only the identification of microbial communities but also the expression-based evaluation of novel resistance mechanisms, which is critical for understanding the complex dynamics of ARGs transmission among bacterial populations in aquaculture systems [67].

Freshwater aquaculture systems represent significant environments where metagenomic studies have been conducted extensively. For instance, Hemamalini et al., [65] presented a critical review that surveyed the sources of antibiotic resistance, the impact of antibiotic residues, and the overall utility of metagenomics in detecting ARGs in freshwater aquaculture systems. In alignment with these findings, Wang et al., [68] focused on coastal industrial mariculture systems, where metagenomic analysis revealed the presence of a wide spectrum of ARGs, indicating that both conventional and recirculating aquaculture systems can serve as reservoirs for resistance determinants. Such differential profiling emphasizes that aquaculture environments are not homogenous and that metagenomics can assist in tailoring monitoring strategies specific to system type [65,68].

#### 3.2.3. The Trend Topics

**Figure 8:**
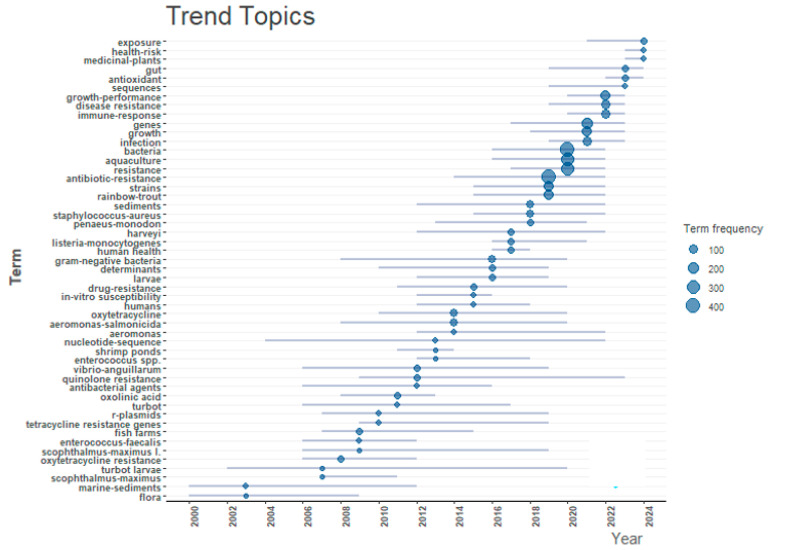
Trend Analysis based on scientific terms of ABA documents (2000–2025). Each dot represents a specific term’s appearance in the literature, and the dot’s size indicates the term’s frequency of occurrence. The horizontal lines show the active period for each term. ABA: “antibiotic resistance”, “bacteria”, and “aquaculture”.

The analysis of trend topics over the period from 2000 to 2024 provides a comprehensive view of the evolving research focus in the ABA field. The data indicate a marked increase in research activity related to antimicrobial resistance (AMR), particularly from 2010 onwards (Figure 8). Terms such as “antibiotic resistance”, “drug-resistance”, “tetracycline resistance genes”, and “multi-drug resistance” have shown a pronounced rise in frequency, reflecting the growing global concern over resistant pathogens in both environmental and clinical settings. Notably, terms such as “aquaculture”, “rainbow trout”, and “sediment” suggest an increasing research emphasis on environmental reservoirs and the role of aquaculture in the dissemination of antibiotic-resistant genes. Antibiotic applications in intensive aquaculture systems have been shown to cause genetic adaptations in microbial communities, leading to the selection and persistence of diverse resistance determinants. For example, research performed in high-intensity catfish production systems showed that therapeutic use of oxytetracycline was associated with genetic shifts within bacterial populations, contributing to an increased number of resistant strains [69]. Such results underscore the dual threat presented by aquaculture practices: aquatic ecosystems become reservoirs for resistance genes. Fish and other aquacultured animals may act as vectors, transferring these genes to human pathogens in clinical settings [69,70]. This aligns with the one wellbeing perspective, which emphasizes the interconnectedness of animal, human, and environmental health.

Based on our results, the consistent appearance and increasing frequency of bacterial genera such as “*Vibrio*”, “*Enterococcus*”, “*Aeromonas*”, “*Staphylococcus aureus*”, and “*Listeria monocytogenes*” underscore their significance as both pathogens and as indicators in AMR research (Figure 8). These pathogens are significantly important not only because of their pathogenic potential but also due to their capacity to serve as indicators for the emergence and dissemination of AMR in aquatic environments [71,72].

*Enterococcus* spp. have been frequently detected in aquaculture-related samples, including fish feeds and aquatic animals, positioning them as both opportunistic pathogens and indicators of fecal and environmental contamination [73,74]. Their ability to harbor and transfer AMR genes, often mediated by mobile genetic elements such as plasmids and transposons, further enhances their significance in AMR research [73]. Such observations support the utility of *Enterococcus* spp. as a model organism for monitoring resistance trends across different environmental and clinical sectors. In this regard, the frequent isolation of these bacterial species from aquaculture products and their surrounding environments provides critical insight both into disease outbreaks in aquatic organisms and the underlying AMR dynamics.

Similarly, *Aeromonas* spp. are abundant in aquatic environments and cause a variety of fish diseases. They are also considered important carriers of resistance determinants, and recent research has emphasized that the occurrence of AMR in these species is intricately linked to the practices and antimicrobial usage within aquaculture systems [75]. Their dual role as pathogens and reservoirs of resistance underscores the interplay between antimicrobial application in aquaculture and the broader emergence of AMR.

In addition, *Staphylococcus aureus* has been identified in aquaculture environments and is notable for its strong resistance mechanisms and classification among priority pathogens in the global AMR situation [76]. Although *S. aureus* is more traditionally associated with human and land-based animal infections, its detection in aquatic environments expands our understanding of AMR dissemination pathways, indicating cross-sectoral contamination and the need for integrated surveillance.

Furthermore, *Listeria monocytogenes*, although historically was connected with foodborne outbreaks, is increasingly being observed in aquatic products, even though its occurrence is less frequently recorded compared to *Vibrio* spp. and *Aeromonas* spp. in aquaculture. However, it raises a significant concern as it acts both as a direct pathogen to humans and an indicator of microbial contamination and antimicrobial selection pressure in aquatic systems [71]. This expanding detection warrants a multidisciplinary approach in which aquaculture AMR surveillance includes pathogens traditionally examined in the context of terrestrial food safety.

#### 3.2.4. The Current Situation of Antibiotic Resistance in Aquaculture

Based on our comprehensive review of the latest published scientific articles on the antibiotic-resistant bacteria in aquaculture (2024–2025) (Table 3), different aquatic environments in several countries provide important insights into the global extent and diversity of antimicrobial resistance (AMR) in the aquatic systems. The data reveal a worrying pattern of widespread resistance among both environmental and aquaculture-associated bacterial species, highlighting the role of water bodies as critical reservoirs and vectors of resistance genes. A wide range of antibiotic classes are involved, including macrolides, quinolones, tetracyclines, beta-lactams, sulfonamides, and aminoglycosides, among others. Notably, resistance to multiple antibiotics was frequently observed, suggesting the presence of multidrug-resistant organisms. For example, bacterial isolates from China’s marine and freshwater systems showed high resistance to more than 30 antibiotics, and studies from Bangladesh revealed high levels of resistance in *E. coli*, *Salmonella* spp., and *Shigella* spp. in tilapia (*Oreochromis niloticus*) and Sea urchin (*Tripneustes gratilla*) (Table 3).

### 3.3. The Alternative Approaches

**Figure 9:**
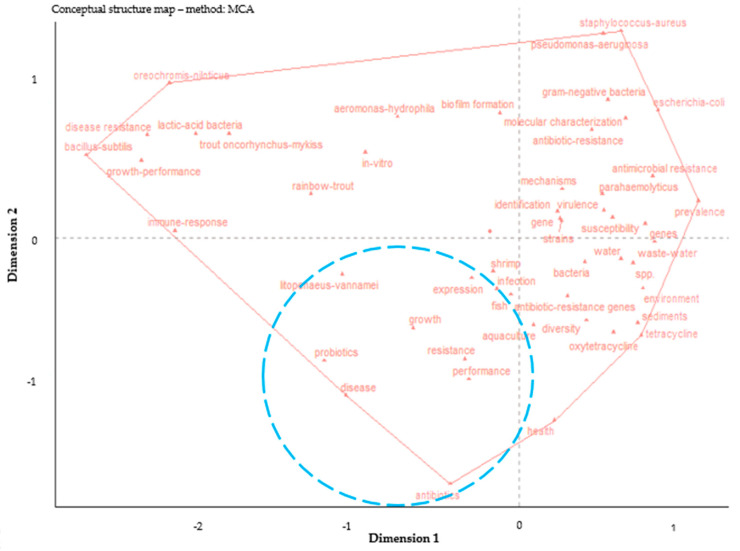
The conceptual structure mapping generated by multiple correspondence analysis of ABA documents based on WoS database (2000–2025). ABA: “antibiotic resistance”, “bacteria”, and “aquaculture”.

Based on our bibliometric analysis, the conceptual structure map (Figure 9) organizes key terms into thematic regions according to their conceptual similarity and frequency of co-occurrence. The left side of the map includes topics such as lactic-acid bacteria, growth performance, trout (*Oncorhynchus mykiss*), and immune response, reflecting a focus on aquaculture productivity and health enhancement. The bottom section included key terms like probiotics, aquaculture, performance, expression, and resistance, suggesting an integrated research on disease management strategies and productivity of aqaculture species.

The integration of probiotics (the beneficial microbes) into aquaculture practices has received a remarkable research attention due to its potential to enhance disease management, growth performance, gut microbiota, digestibility-related enzymes, and overall performance of cultured aquatic animals [28,92,93,94]. In the intensive aquaculture systems where overcrowding and stress occur, fish became more susceptible to infections, the strategic use of probiotics offered an effective alternative to traditional antimicrobials, reducing the reliance on antibiotics, and thereby mitigating the emergence of antimicrobial resistance (AMR) [13,28].

An integrated research approach that combines aspects of disease management with productivity outcomes involves evaluating probiotics for their ability to modulate immune gene expression and physiological performance. For example, several studies have demonstrated that the dietary *Bacillus* spp. supplements could improve nutrient digestibility and growth performance, as well as enhance the innate immunity of fish, as indicated by elevated levels of immune-related enzymes and cytokine expression [95,96]. Similarly, the application of probiotics as water additives has been shown to stimulate both local and systemic immune responses in tilapia (*Oreochromis niloticus*), contributing to higher survival rates and improved overall performance against common pathogens [97,98]. These findings underscore the dual role of probiotics in enhancing aquaculture productivity while simultaneously serving as a disease control strategy.

Furthermore, integrated research has revealed that the benefits of probiotics extend to stress modulation and improvement in feed utilization. Enhanced feed conversion ratios and growth performance have been documented when probiotics are incorporated into feed, as they promote a balanced gut microbiota and facilitate better absorption of nutrients [99]. The modulation of the host’s immune system, such as the upregulation of key immune genes, further contributes to disease resistance and improved stress resilience, particularly under challenging environmental conditions [100]. This integrated approach not only supports the economic performance of the aquaculture industry by enhancing production but also contributes to environmental sustainability by reducing the need for chemical interventions [101,102].

It is clear from the literature that the benefits of probiotics in aquaculture are multifaceted. They include improved growth performance, enhanced immune modulation, and greater disease resistance (Table 4). All of which are essential for sustainable aquaculture practices. Moreover, by alleviating the emergence of AMR through reduced antibiotic use, probiotics serve as an integral component of an environmentally sound and economically viable strategy in modern aquaculture [103,104,105]. Integrated research that further explores the molecular mechanisms underlying these benefits such as specific gene expression patterns in response to probiotic administration remains a promising avenue for innovation in the field of aquaculture probiotics [106,107].

**Figure 10:**
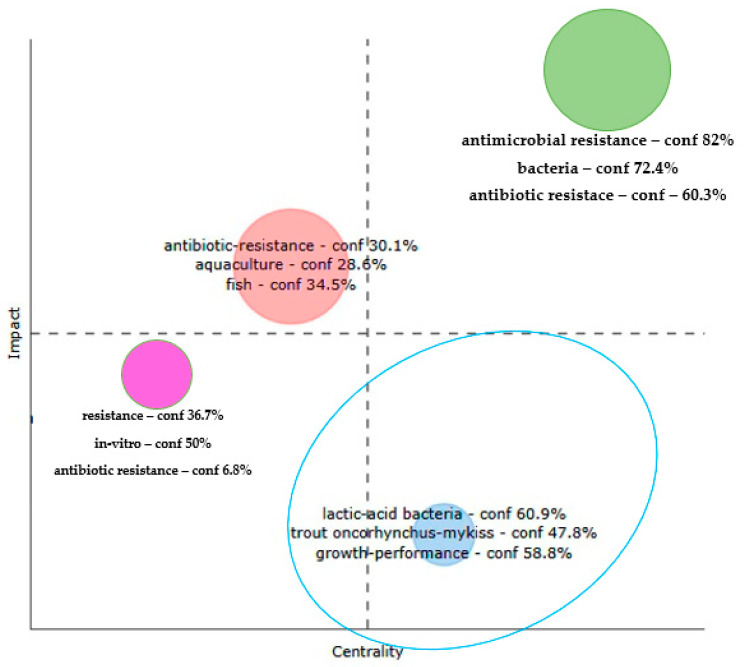
Thematic map of research clusters generated by multiple correspondence analysis of ABA documents based on WoS database 2000–2025. ABA: antibiotic resistance, bacteria, and aquaculture.

On the other hand, the multiple correspondence analysis of ABA documents based on WoS database (2000–2025) (Figure 10) showed that the lower-right quarter of the thematic map represents emerging themes that are characterized by relatively high internal coherence (impact) but lower centrality, indicating limited integration with the broader research network. Within this quarter, the dominant terms “lactic-acid bacteria”, “trout (*Oncorhynchus mykiss*)”, and “growth performance” provide important insights into the evolving focus of antimicrobial resistance research in aquaculture.

The term “lactic-acid bacteria” (confidence: 60.9%) stands out as a key emerging topic. This reflects the growing scientific interest in the use of probiotics as a sustainable alternative to antibiotics in aquaculture. Lactic acid bacteria (LAB) are widely recognized for their antimicrobial properties, immunomodulatory effects, and ability to enhance gut health, which can collectively reduce the need for therapeutic antibiotic use. Their inclusion in this quadrant suggests that although this area is gaining traction, it remains somewhat disconnected from the central discourse on resistance mechanisms and bacterial ecology, warranting greater interdisciplinary integration.

LAB-based probiotics have been extensively studied for their potential to improve feed utilization and growth performance. Research demonstrates that LAB can enhance nutrient digestion by synthesizing extracellular enzymes, which facilitate better feed absorption and ultimately lead to improved growth metrics in species such as Nile tilapia (*Oreochromis niloticus*) and common carp (*Cyprinus carpio*) [108]. In addition to these nutritional benefits, LAB have been linked to the modulation of gene expression related to immune responses and stress resilience, reinforcing their role in integrated disease management strategies in aquaculture and also contributing to reduced spread of antimicrobial resistance [109].

**Table 4:** Effectiveness of probiotics in aquaculture as documented in the reviewed ABA documents (2024–2025). ABA: “antibiotic resistance”, “bacteria”, and “aquaculture”.

Country	Probiotic/Aquaculture Species	Main Results	Date	Ref.
China	*Endozoicomonas* spp. Clam (*Meretrix petechialis*)	*Vibrio* spp. loads ↓ Enhancing inflammatory response (NF-kappa B) ↑ Oxidative response (ROS metabolism) ↑	March 2025	[110]
China	*Rhodopseudomonas palustris* (3 × 10^8^ CFU/mL) and composite probiotics (5 × 10^8^ CFU/mL) with ratio of 1:1 Sea urchin, *Strongylocentrotus intermedius*	Immune indicators (ACP, AKP, LZM) ↑ Digestive enzyme ↑ Microbiota diversity ↑ Expression pf immune related genes ↑	Feb. 2025	[111]
China	*Lacticaseibacillus rhamnosus* FS3051, and *L. reuteri* FS3052 Grey mullet, *Mugil cephalus*	Resistance to *Nocardia seriolae* ↑ Secretion of hydrolytic enzymes ↑ weight gain, feed efficiency, and growth rate (%) ↑ Regulation of IL-8, TNF-alpha, IL-1 beta, IFN-gamma, and MHCI ↑ The abundance of *Mycoplasma* and *Rhodobacter* in the gut microbiome ↓	Nov. 2024	[112]
China	*Bacillus velezensis* BV1704-Y Zebrafish (*Danio rerio*)	*Aeromonas hydrophila* ↓ Mortality ↓ Expression IL-1 beta, TNF-alpha, IL6 ↓ Abundance of *Cetobacterium* ↓	Sept. 2024	[113]
Poland	*Levilactobacillus brevis* pikeperch (*Sander lucioperca* L.)	Inhibition of *Aeromonas hydrophila*, *A. salmonicida*, *Acinetobacter junii*, *Pseudomonas fluorescens* ↑	Sept. 2024	[114]
Malaysia	Probiotics tilapia (*Oreochromis* sp.)	Weight gain ↑ Feed conversion ratio ↓ Innate immunity ↑ pathogenic challenge ↑	Feb. 2024	[115]
China	*Bacillus subtilis* JSHY-K3 shrimp *Penaeus vannamei*	Resistance against *Vibrio parahaemolyticus* ↑ Acute hepatopancreatic necrosis ↓	August 2024	[116]
Thailand	Kratom leaf extract (*Mitragyna speciose*) Nile tilapia (*Oreochromis niloticus*)	Expression of IL6, IL8, NF-kB, IFN gamma, TNF alpha, CC-chemokine, MHC-II beta, CD4, TCR beta, IgT, IgM, IgD ↑ Resistance against *Edwardsiella tarda* ↑	Sept. 2024	[117]
USA	*Lactococcus lactis* (MA5) catfish (*Ictalurus punctatus* × *I. furcatus*)	Inhibition of catfish bacterial pathogens ↑ Antibiotic susceptibility ↑	Oct. 2024	[118]
Spain	Lactic acid bacteria (LAB) LAB and pathogens were obtained from mucus of *Oncorhynchusmykiss* and *Salmo trutta*	Susceptibility to oxytetracycline ↑ Antagonistic against *Aeromonas salmonicida* subsp. *salmonicida*, *Carnobacterium maltaromaticum*, *Vagococcus salmoninarum, Yersinia ruckeri, Lactococcus garvieae*, and *Vibrio jasicida* ↑	Jan. 2024	[119]
Malaysia	*Lactobacillus plantarum* L20 and *Sargassum polycystum*-supplemented diet Black tiger shrimp (*Penaeus monodon*)	Resistant against Acute hepatopancreatic necrosis caused by *Vibrio parahaemolyticus* ↑ Immune response ↑	Feb. 2024	[120]
Colombia	*Cetobacterium* sp.* nov. C33* Nile Tilapia (*Oreochromis niloticus*)	Gastrointestinal adaptability ↑ Ability to adhere to intestinal epithelial cells ↑ Producing antimicrobial substances ↑	Dec. 2023	[121]
Indonesia	*Serratia marcescens* Van80 UB3 and *Shewanella algae* A3 White Shrimp *(Litopenaeus vannamei)*	Digestive enzyme ↑ Growth-related genes ↑ Improved immunity ↑	May 2025	[122]

“↑”: Increase/upregulate, “↓”: Decrease/downregulate.

## 4. Conclusions

The dissemination of antibiotic-resistant bacteria in aquaculture (ABA) represents a growing threat to the aquatic environments and human, primarily caused by the extensive use of antibiotics, which exerts selective natural pressure and leads to the proliferation of resistant bacterial strains. In addition, extensive use of antibiotics facilitates the horizontal transfer of antibiotic resistance genes (ARGs) among various bacterial communities through mobile genetic elements and plasmids. These genetic exchanges can occur in biofilms or sediment-associated niches of aquaculture systems, where conditions foster the persistence and spread of ARGs even in the absence of antibiotic application.

Through a comprehensive bibliometric analysis of 1869 publications (scientific papers, book chapters, and conference proceedings) from 2000 to 2025, the study identifies significant growth in research activity, driven primarily by contributions from China, India, and the USA. The analysis reveals that international collaborations have become increasingly important in tackling this global health issue, with countries like UK, Belgium, and Thailand demonstrating high levels of cross-border research engagement.

The results illustrate a concerning trend of multidrug-resistant (MDR) bacteria and a growing prevalence of the ARGs in aquaculture systems. Pathogenic bacteria such as *Vibrio*, *Aeromonas*, *Enterococcus*, and *E. coli* are recurrently identified as vectors of resistance, often linked to intensive antibiotic use in aquaculture practices. Emerging keywords and network analyses show a shift toward molecular approaches, especially metagenomics, which allow for in-depth profiling of resistance mechanisms and microbial dynamics in aquatic ecosystems.

Importantly, the review underscores the critical need for alternative, sustainable strategies to mitigate antibiotic use. Probiotics, especially lactic acid bacteria (LAB), have emerged as promising candidates due to their immunomodulatory effects, growth-promoting potential, and role in reducing pathogen loads. The study showcases various experimental applications of probiotics across fish and shellfish species, highlighting improvements in immune response, gut health, and disease resistance.

The findings stress the urgency of adopting a multidisciplinary and globally coordinated approach to combat antibiotic resistant bacteria in aquaculture. Future efforts should prioritize innovation in non-antibiotic disease control methods, expand the use of molecular surveillance tools, and establish international monitoring frameworks. Addressing antimicrobial resistance is not only a matter of aquaculture sustainability, but also a central aspect of the One Health agenda that links animal, human, and ecosystem wellbeing.

## Figures and Tables

**Table 1:** The top 20 producing countries of ABA documents (2000–2025) based on the WoS database. ABA: “antibiotic resistance”, “bacteria”, and “aquaculture”.

Rank	Country	Papers	% of 1869	SCP *	MCP	MCP Ratio
1	China	520	27.82	442	78	15.00
2	India	203	10.86	170	33	16.26
3	Malaysia	77	4.12	46	31	40.26
4	USA	76	4.07	50	26	34.21
5	Korea	69	3.69	52	17	24.64
6	Spain	52	2.78	32	20	38.46
7	United Kingdom	46	2.46	24	22	47.83
8	Brazil	42	2.25	37	5	11.90
9	Chile	42	2.25	34	8	19.05
10	Italy	39	2.09	30	9	23.08
11	Thailand	37	1.98	14	23	62.16
12	Egypt	36	1.93	20	16	44.44
13	Canada	33	1.76	23	10	30.30
14	Portugal	33	1.76	24	9	27.27
15	Turkey	33	1.76	30	3	9.09
16	France	31	1.66	22	9	29.03
17	Belgium	29	1.55	14	15	51.72
18	Mexico	28	1.50	22	6	21.43
19	Indonesia	27	1.44	22	5	18.52
20	Japan	27	1.44	14	13	48.15

* SCP: single country publications, MCP: multiple country publications.

**Table 2:** Top 20 cited documents on ABA topic (2000–2025). ABA: “antibiotic resistance”, “bacteria”, and “aquaculture”.

Rank	Paper	* TC	TC per Year	Publisher	Ref.
1	CABELLO FC, 2006, ENVIRON MICROBIOL	1607	80.35	Wiley	[47]
2	VERSCHUERE L, 2000, MICROBIOL MOL BIOL REV	1468	56.46	American Society for Microbiology	[48]
3	LUIS MARTINEZ J, 2009, ENVIRON POLLUT	1365	80.29	Elsevier	[49]
4	NIKAIDO H, 2009, ANNU REV BIOCHEM	1100	64.70	Elsevier	[50]
5	LIU X, 2017, ENVIRON POLLUT	709	78.78	Elsevier	[16]
6	SAPKOTA A, 2008, ENVIRON INT	625	34.72	Elsevier	[51]
7	ZAINAB SM, 2020, WATER RES	624	104.00	Elsevier	[52]
8	CABELLO FC, 2013, ENVIRON MICROBIOL	590	45.38	Wiley	[53]
9	PRUDEN A, 2013, ENVIRON HEALTH PERSPECT	579	44.54	National Institute of Env. Health Sciences	[54]
10	GOTHWAL R, 2015, CLEAN-SOIL AIR WATER	562	51.09	Wiley	[55]
11	GAO P, 2012, WATER RES	532	38.00	Wiley	[11]
12	DEFOIRDT T, 2011, CURR OPIN MICROBIOL	519	34.60	Elsevier	[56]
13	WATTS JEM, 2017, MAR DRUGS	438	48.67	MDPI	[13]
14	ZOU S, 2011, ENVIRON POLLUT	413	27.53	Elsevier	[57]
15	VIECO-SAIZ N, 2019, FRONT MICROBIOL	366	52.28	Frontiers Media S.A.	[58]
16	HEUER OE, 2009, CLIN INFECT DIS	365	21.47	Oxford University Press	[59]
17	KYU-SONG S, 2014, FISH SHELLFISH IMMUNOL	338	28.17	Elsevier	[60]
18	EL-SAADONY MT, 2021, FISH SHELLFISH IMMUNOL	336	67.20	Elsevier	[61]
19	ROMERO J, 2012, HEALTH AND ENVIRONMENT IN AQUACULTURE	335	23.93	IntechOpen	[62]
20	SANTOS L, 2018, INT J ANTIMICROB AGENTS	326	40.75	Elsevier	[37]

* “TC”: Total citations.

**Table 3:** Prevalence of antibiotic-resistant bacteria, resistance genes, and associated antibiotics in aquatic environments based on the most recent ABA documents (2024–2025) from WoS. ABA: “antibiotic resistance”, “bacteria”, and “aquaculture”.

Country	Aquatic Environment/Species	Antibiotic-Resistant Bacteria	Antibiotic	Resistant Genes/Toxic Proteins	Date	Ref.
Turkey	Marmara Sea	*Microbacterium istanbulense* sp. nov., *M. bandirmense* sp. nov., *M. marmarense* sp. nov	Macrolide	Erm23S_rRNA methyltransferase	March 2025	[77]
Chile	Salmon Farms (2 fish farms)	*Gammaproteobacteria*	Phenicol	*floR*	Jan. 2025	[78]
Bangladesh	Two fish lakes and three rivers	*Shigella flexneri* *Escherichia ferguso* *Proteus mirabilis* *Enterobacter quasiroggenkampii*	Ciprofloxacin Ceftriaxone Ampicillin	**-**	Jan. 2025	[79]
China	Three traditional fish ponds, Tai Lake, Zhejiang	*Proteobacteria* *Cyanobacteria* *Actinobacteriota* *Bacteroidota* *Verrucomicrobiota*	Quinolone Chloramphenicol	*tetA*, *tetB*, *tetC*, *tetG*, *tetM,* *sul1*, *qnrB*, *ermF*, *cat1* and *intI1*	Jan. 2025	[80]
China	*Mastacembelus armatus* Strain YY001	*Plesiomonas* *shigelloides*	Oxacillin Norfloxacin Tetracycline	*emrD*, *macB*, *catB*, *ksgA*, *tolC*, *MdfA*, *sul1*, *bacA* and *DegP*	Dec. 2024	[81]
Israel	Sea urchin (*Tripneustes gratilla*)	*Vibrio* spp. (Red-Spotting Diseases)	Tetracycline Penams	*adeF* *CRP*	Nov. 2024	[82]
China	Internal organs of Largemouth Bass, *Micropterus salmoides*	*Edwardsiella piscicida*	Ciprofloxacin (96.25%) Sulfonamides (60–63%) Thiamphenicol (56.2%) Florfenicol (43.75%) Enrofloxacin (32.50%) Doxycycline (16.25%) Flumequine (1.25%)	-	Nov. 2024	[83]
China	Sea turtles	Nine *Vibrio* spp.	Nitrofurans Aminoglycosides	*merA*	Nov. 2024	[84]
India	Shrimp ponds	*Vibrio* spp. *Exiguobacterium* spp. *Microbacterium* spp.	Oxytetracycline Ciprofloxacin Co-trimoxazole Chloramphenicol Erythromycin	*tetA* (44.0%) *sul1* (11.8%)	Jan. 2025	[85]
China	Diseased yellowfin seabream, *Acanthopagrus* *latus* (VH-AQ-SCAU-GZ23)	*Vibrio harveyi* (VH-AQ-SCAU-GZ23)	Thermolysin Tetracyclines Aminoglycosides Quinolones Macrolides Other antibiotics.	*TLH*	Jan. 2025	[86]
China	Seawater (South China Sea)	*Proteobacteria* *Cyanobacteria*	Aminoglycoside Tetracycline fluoroquinolone antibiotics accounted for	77.3–88.6% of the total ARGs in seawater were resistant to these antibiotics.	Sept. 2024	[87]
India	Oscar fish (*Astronotus ocellatus*)	*Edwardsiella* *tarda*	Multidrug	*E. tarda* caused 100% mortality within 240 h with 6.99 × 10^6^ CFU/fish	Jul. 2024	[88]
China	Coastal ecosystems (Sediments from seagrass meadow)	*Halioglobus* spp. *Zeaxanthinibacter* spp. *Aureitalea* spp.	30 antibiotics were detected with total of 99.35–478.02 μg/kg Tetracyclines were the most common.	22 ARG were identified. Multidru resistance genes and *ranA* were the most common.	Jul. 2024	[89]
Bangladesh	Tilapia fish (*Oreochromis niloticus*) for sale at retail markets in Dhaka city	*E. coli* (92%) *V. cholerae* (62%) *E. coli* ESBL (48%) *Salmonella* spp. (24%) *Salmonella* spp. *Shigella* spp. *Cryptosporidium* spp.	β-lactamase concentration was 2.3 ± 0.8 log_10_ CFU/g in *E. coli*	40% of *Salmonella* (*stn*, *bcfC*, *avrA*, *sodC1*, *ssaQ*)	April 2024	[90]
Egypt	Fish farm in an earthen pond, Damietta.	*V. alginolyticus* (205 isolates) *V. fluvialis* (87 isolates)	Amoxicillin Erythromycin	-	April 2024	[91]

“-” value is not available.

## Data Availability

Data supporting this study’s findings are available from the corresponding author upon request.
